# Supplementary material for: A comprehensive and easy-to-use multi-domain multi-task medical imaging meta-dataset
Source: Sci Data. 2025 Apr 19;12:666. doi: 10.1038/s41597-025-04866-4 (PMC12009356; doi:10.1038/s41597-025-04866-4)
Supplement: Supplementary file 1 — Training algorithms for validation baselines [file 41597_2025_4866_MOESM1_ESM.pdf]

Supplementary material for: A comprehensive and easy-to-use multi-domain multi-task medical imaging meta-dataset

Stefano Woerner[1], Arthur Jaques[1], and Christian F. Baumgartner[1,2]

[1]Cluster of Excellence “Machine Learning: New Perspectives for Science”, University of Tübingen, Germany
[2]Faculty of Health Sciences and Medicine, University of Lucerne, Switzerland

Training Algorithms

Multi-domain multi-task pre-training

To address the issue of catastrophic forgetting, we propose a multi-domain multi-task pre-training schedule (which we abbreviate with mm-PT), where the model is simultaneously pre-trained on all datasets in our meta-dataset. This means that the model will be simultaneously trained on multiple heterogeneous domains and tasks. To achieve this, the network is divided into a backbone and a classification head, where the last linear layer of the network is removed to create the backbone, and a linear layer is initialized as the head for each task. The algorithm for mm-PT involves randomly sampling batches from each of the tasks and training the model on these batches. The appropriate head and loss function is used for each batch, as illustrated in algorithm 1. When fine-tuning to the target task, a new head appropriate for the target task is attached to the backbone.

Algorithm 1 Multi-domain Multi-task Pre-training

Require: Set T of tasks, Datasets D_t for tasks $t \in T$, Model f with parameters θ, Classification heads l_t for $t \in T$

```
while training has not converged do
    t ← Sample a task t from T with probability |D_t| / Σ_{j∈T} |D_j|
    B ← Sample a batch from dataset D_t
    θ ← θ − ∇_θ 𝓛_t(l_t(f(B; θ)))
end while
return θ
```

Multi-domain multi-task meta-learning

The appearance of medical images can vary significantly depending on the imaging modality used. Furthermore, the nature of tasks involved in medical image analysis varies considerably, encompassing a range of classifications such as binary, multi-class, or multi-label, and differing in the number of target labels or classes required.

To enable meta-learning in a multi-domain multi-task setting, we propose a simple extension of MAML [1] that can learn from a diverse set of tasks prior to fine-tuning on the target task. Although MAML can be applied to almost any neural network architecture, the assumption of a constant architecture becomes an issue when training with a set of diverse tasks: if tasks have diverse targets, at least the output layer has to change. We have investigated attaching new per-task classification layers as an extension of MAML and how to initialize them in [2]. Initialising the weights of the classification layer with zeros mitigates the performance drop experienced with the default Kaiming initialisation. We therefore use this strategy as our default initialization scheme for the task-specific classification heads. Additionally, in each episode we use the loss function which is appropriate for the respective task.

The proposed Multi-domain Multi-task MAML (mm-MAML) is trained simultaneously on all datasets in our meta-dataset by sampling few-shot tasks at random from each of the tasks and using the appropriate head and loss function. At meta-test time, i.e. when fine-tuning to the target task, a new head and loss function is simply created for each target task, exactly as described in the previous section on transfer learning. The training algorithm is described in Algorithm 2.

Algorithm 2 Multi-Domain Multi-Task MAML

Require: Set T of tasks, Datasets D_t for tasks $t \in T$, Model f with parameters θ and meta-parameters φ, The number of inner steps I

while training has not converged **do**
    $t \leftarrow$ Sample a task t from T
    $C \leftarrow$ Sample a set C of classes from $\mathscr{P}(C_t)$
    $l \leftarrow$ create a classification head l for the task t with classes C
    $S \leftarrow$ Sample a support set from dataset D_t
    $Q \leftarrow$ Sample a query set from dataset D_t
    $\theta \leftarrow \varphi$
    for $i \in \{1 \dots I\}$ **do**
        $\theta \leftarrow \theta - \nabla_\theta \mathscr{L}_t(l(f(S;\theta)))$
    end for
    $\varphi \leftarrow \varphi - \nabla_\varphi \mathscr{L}_t(l(f(Q;\theta)))$
end while
return θ
